# Multimorbidity in a cohort of middle-aged women: Risk factors and disease clustering

**DOI:** 10.1016/j.maturitas.2020.04.016

**Published:** 2020-07

**Authors:** Juan E. Blümel, Rodrigo M. Carrillo-Larco, María S. Vallejo, Peter Chedraui

**Affiliations:** a Departamento De Medicina Interna Sur, Facultad De Medicina, Universidad De Chile, Santiago De Chile, Chile; b Department of Epidemiology and Biostatistics, School of Public Health, Imperial College London, London, United Kingdom; c Clínica Quilín, Facultad De Medicina, Universidad De Chile, Santiago De Chile, Chile; d Instituto De Investigación e Innovación En Salud Integral, Facultad De Ciencias Médicas, Universidad Católica De Santiago De Guayaquil, Guayaquil, Ecuador; e Facultad De Ciencias De La Salud, Universidad Católica “Nuestra Señora De La Asunción”, Asunción, Paraguay

**Keywords:** Multimorbidity, Women, Aging, Ageing, Elder, Obesity, Risk factors

## Abstract

**Objective:**

We aimed to evaluate which risk factors in middle-aged women are associated with higher risk of multimorbidity in older age.

**Study design:**

We conducted a prospective cohort study from 1990 to 1993 in Santiago de Chile, Chile among women aged 40–59 (at baseline). Diagnosed illnesses were retrieved from national health records in 2020.

**Main outcome measures:**

: Clinical and laboratory evaluation was conducted.

**Results:**

1066 women were followed-up for a mean of 27.8 years, after which 49.7% presented multimorbidity. These women, as compared with those without multimorbidity, were more likely at baseline to have had obesity (20.4% vs. 8.6%, *p* < 0.001); be post-menopause (47.2% vs. 40.5%; *p* < 0.03); have jobs that did not require a qualification (74.2% vs. 56.0%, *p* < 0.001); arterial hypertension (19.8% vs 14.4%, *p* < 0.018); lower HDL-cholesterol (51.3 ± 12.9 vs. 53.6±12.7 mg/dL, *p* < 0.005); and higher triglyceride levels (136.0 ± 65.0 vs. 127.0 ± 74.0 mg/dL, *p* = 0.028). Hypertension was associated in 22.0% of women with diabetes, in 20.9% with osteoarthritis and 14.0% with depression. Osteoarthritis was also associated with diabetes mellitus (8.3%) and depression (7.8%). Diabetes mellitus, in addition to hypertension and osteoarthritis, was associated with depression (6.4%). In a logistic regression model, we observed that obesity in middle-aged women was the strongest risk factor for multimorbidity in the elderly (OR: 2.48; 95% CI, 1.71–3.61), followed by having a job that did not require a qualification (OR: 2.18; 95% CI, 1.67–2.83) and having a low HDL-cholesterol level (OR: 1.31; 95% CI, 1.02–1.68).

**Conclusions:**

Multimorbidity was highly prevalent in this older female population. Obesity in middle-aged women was the strongest risk factor for multimorbidity at older age. These results are relevant for Chile and other countries with similar population profiles.

## Highlights

• Multimorbidity is highly prevalent, especially among those aged 65 and above.
• Globally, there is a lack of evidence on long-term risk factors for multimorbidity.
• This work presents a cohort of women in Chile followed for 28 years.
• Arthrosis, hypertension, diabetes, and depression were frequent components of multimorbidity.
• Obesity in middle-aged women was the strongest risk factor for multimorbidity.

## 1 Introduction

A large proportion of the global population, especially those aged 65 and above, is affected by multimorbidity [1]. This has been defined as the co-existence of at least two chronic diseases in the same individual [2]. However, there are broader definitions, such as the one proposed by the European General Practice Research Network, which defines multimorbidity as the combination of a chronic disease with at least one other chronic or acute illness, or a socio-economic or biological risk factor [3]. Regardless of the definition, multimorbidity implies increased hospitalizations or mortality rates, fragility, depression, polypharmacy, and worse overall quality of life; these impaired outcomes increase the burden on healthcare systems and related expenditures [4].

Multimorbidity has a negative impact on the workforce, affecting productivity and increasing absenteeism. Employability also decreases with multimorbidity because employers tend not to hire those who are ill. All this also affects the quality of life of workers and deteriorates the overall country economy [5].

As populations of the world age rapidly, multimorbidity is becoming a major concern in public health. The Academy of Medical Science (London, UK) has positioned multimorbidity as a priority for global health research. In most high-income countries, multimorbidity is considered the norm and not the exception; however, it also appears to be increasingly prevalent in low- and middle income countries [6].

A non-modifiable risk factor for multimorbidity is aging. However, this only explains part of the increasing multimorbidity prevalence seen in recent years; thus, other factors such as healthcare and socio-economic profiles are also responsible for the substantial rise of multimorbidity burden [7].

Much of the available evidence has focused on describing the prevalence and patterns of multimorbidity, though the long-term determinants, i.e. risk factors associated with multimorbidity incidence, have been poorly examined. Consequently, we aimed at evaluating which risk factors in middle-aged women were positively associated to a higher risk of multimorbidity after, approximately, 28 years of follow-up.

## 2 Materials and methods

We conducted a prospective cohort study in the *Servicio de Salud Metropolitano Sur* (*Hospital Barros Luco*, Santiago de Chile, Chile), between October 1990 and March 1993. For this, we invited all women who attended the service for preventive healthcare checkups, which is mandatory for public servants on an annual basis. Clinical evaluation and questionnaire applications were carried out by experienced healthcare professional**s** (at least ten years).

A total of 1229 women were invited at baseline of which 1197 (97.4%) accepted to participate. We excluded 38 participants because of missing data and 93 who died before age 70. Women who died after age 70 were included because multimorbidity prevalence was similar between them and those who were still alive (45.6% vs. 50.2%, *p* = 0.399). Final statistical analysis included 1066 women followed-up for a mean of 27.8 years (range: 26–29 years).

Based on questionnaires, clinical and anthropometric assessments, we collected the following information: identification number, date of health control, date of birth, age, occupation, years of education, height, weight, menopausal status, plasma glucose levels and the self-reported use of medication for diabetes/hypertension, systolic/diastolic blood pressure and total physical activity (minutes per week).

Total serum cholesterol and triglycerides levels were assessed with enzymatic calorimetric methods (Sigma, Sigma Chemical Co., St. Louis, Missouri, USA). High-density lipoprotein cholesterol (HDL-C) was precipitated with magnesium sulphate and dextran (Sigma, Sigma Chemical Co.). Intra-assay and inter-assay coefficient variations for total cholesterol, HDL-C and triglycerides were 1.6%, 3.9% and 3.9% and 4.2%, 4.6% and 3.9%, respectively. Low-density lipoprotein cholesterol (LDL-C) was calculated with the Friedewald formula. Fasting glucose was measured with calorimetric methods (Hexokinase; Sigma Chemical Co.), with an intra-assay and inter-assay coefficient variations of 0.7% and 1.2%, respectively.

At baseline the following variables were considered: older age (≥50 years); obesity (body mass index ≥30 kg/m^2^); postmenopausal status (≥12 months of amenorrhea); sedentarism (≤150 min of physical activity per week); unqualified jobs (and lacking higher education); type 2 diabetes mellitus (fasting blood glucose ≥126 mg/dL in two measurements or ≥200 mg/dL at 2 h after challenge with 75 g of glucose and/or the use of hypoglycemic drugs); hypertension (systolic/diastolic arterial blood pressure ≥ 140/90 mmHg and/or the use of antihypertensive drugs); hypercholesterolemia (total serum cholesterol ≥200 mg/dL); low HDL-C (≤50 mg/dL); and high LDL-C (≥130 mg/dL).

In the year 2020, using the national identification number we looked for the participants in the national vital registries to ascertain vital status: dead or alive and date and cause of death when applicable. Non-fatal outcomes, i.e. newly diagnosed illnesses, were retrieved from the SIGGES (*Sistema de Gestión de Garantías Explícitas de Salud*) [8]. These diagnoses are based on widely accepted international criteria (i.e. the diagnosis of depression is based on DSM-IV criteria). This system compiles disease diagnoses for which all Chilean citizens should receive care for. The National Health Fund, as well as healthcare providers, must secure care for these diseases [9]. Multimorbidity was defined as the co-existence of at least two chronic diseases in the same individual (World Health Organization) [2]. Our study assessed the following diseases: hypertension, arthrosis (hip or knee), type 2 diabetes mellitus, depression, cancer (breast, ovary, uterine cervix or colorectal), acute myocardial infraction, stroke, chronic obstructive pulmonary disease (COPD), chronic kidney disease, Parkinson’s disease and HIV.

### 2.1 Statistical analysis

Statistical analysis was performed with the SPSS (IBM Statistics, version 21.0. Armonk, NY, IBM Corp.). Results are presented as mean ± standard deviations (SD), percentages, odds ratios and confidence intervals. Normality of data distribution was evaluated with the Kolmogorov–Smirnov test, and the homogeneity of the variance with the Levene’s test. According to this, group comparisons for means were assessed with the Student’s *t*-test or the Mann–Whitney *U* test. Percentage differences were evaluated with the chi square test.

Logistic regression analysis was performed for the simultaneous assessment of several variables influencing multimorbidity prevalence (i.e. prevalent cases at baseline were not ascertained nor excluded). For this, multimorbidity was used as the dependent variable for the regression model. Independent variables to be tested in the regression model, all assessed at baseline, were codified as 0 = absence or 1 = presence and included: older age, obesity, postmenopausal status, sedentarism, unqualified job, type 2 diabetes mellitus, hypertension, hypercholesterolemia, low HDL-C and high LDL-C.

Predictors included in the final model were those with at least 20% of significance. For this, we conducted a backward stepwise selection process. Independence of errors assumption was assessed with the Durbin–Watson test. Multi-collinearity was evaluated with the variance inflation factor. The Omnibus (chi-square test) test was used to explore significant differences between groups of predictors. The Hosmer–Lemeshow test was used to evaluate the goodness of fit of the logistic regression model. For all calculations a *p* value of <0.05 was considered statistically significant.

### 2.2 Ethics

The study was approved by a local ethics committee (Southern Metropolitan Health Service, Santiago de Chile, Chile) and was carried out in complete agreement with the Declaration of Helsinki. All patients provided written informed consent.

## 3 Results

Upon follow-up, a 49.7% of women (*n* = 530) met criteria for multimorbidity (Table 1). At baseline, women with and without multimorbidity had a similar age (48.7 ± 5.2 vs. 48.4 ± 5.8 years, *p *= 0.179). Women who later developed multimorbidity, at baseline were more likely to have obesity, in comparison to their peers who did not develop multimorbidity (20.4% vs. 8.6%, *p* < 0.001). Similarly, in the multimorbidity group there were more women who were postmenopausal (47.2% vs. 40.5%; *p* = 0.03), and had unqualified jobs (74.2% vs. 56.0%, *p* < 0.001). Regarding baseline cardio-metabolic risk factors, women who developed multimorbidity had a higher prevalence of hypertension (19.8% vs. 14.4%, p < 0.018), and lower HDL-C (51.3 ± 12.9 vs. 53.6 ± 12.7 mg/dL, *p* < 0.005) and higher triglyceride levels (136.0 ± 65.0 vs. 127.0 ± 74.0 mg/dL, *p* < 0.028).

**Table 1:** Baseline profile of middle-aged women who developed and did not develop multimorbidity.

Variables	Multimorbidity at follow-up		
	No (*n* = 536)	Yes (*n* = 530)	*p* value*
Age at baseline (years)	48.4 ± 5.8	48.7 ± 5.2	0.179^2^
Age in year 2020 (years)^a^	75.9 ± 5.3	76.5 ± 5.0	0.068^1^
Baseline characteristics			
Body mass index (kg/m^2^)	25.0 ± 3.6	26.8 ± 4.2	0.001^2^
Obesity (%)	8.6	20.4	0.001^3^
Postmenopausal status	40.5	47.2	0.031^3^
Sedentarism (%)	92.5	93.0	0.761^3^
Unqualified job (%)	56.0	74.2	0.001^3^
Type 2 diabetes mellitus (%)	2.2	2.5	0.842^4^
Hypertension (%)	14.4	19.8	0.018^3^
Total cholesterol (mg/dL)	222.0 ± 43	221.0 ± 45	0.845^1^
HDL-cholesterol (mg/dL)	53.6 ± 12.7	51.3 ± 12.9	0.005^1^
LDL-cholesterol (mg/dL)	143.7 ± 39.2	142.9 ± 41.1	0.747^1^
Triglycerides (mg/dL)	127.0 ± 74.0	136.0 ± 65.0	0.028^1^

* *p* values as determined with Student’s *t* test^1^; the Mann–Whitney *U* test^2^; the chi-square test^3^; or Fisher’s exact test^4^.

a Including women who died after age 70.

Hypertension was the most prevalent condition upon follow-up, affecting 61.6% of the studied population (Table 2), followed by hip and knee arthrosis (26.1%), type 2 diabetes (25.8%) and depression (17.9%). The rest of the selected illnesses affected less than 8% of the population, and there were no HIV cases (Table 2). Although not significant, women in the age group 60–69 had, in average, 1.31 diseases, while those in the age group 80 or more had 1.63 (Table 2). Similarly, in these age groups, the prevalence of multimorbidity increased from 36.7%to 50.5% (*p* = 0.183).

**Table 2:** Prevalence of the studied diseases at baseline (as of years 1990–1993) and during follow-up (as of year 2020).

Diseases Basal	All women	Age (years)			*p* value*
		40−49 (*n* = 646)	50−59 (*n* = 382)	≥60 (*n* = 38)	
Hypertension (%)	17.1	11.8	24.1	36.8	0.001^1^
Arthrosis (%)	2.9	1.7	4.7	5.3	0.009^2^
Type 2 diabetes mellitus (%)	2.3	1.9	3.1	2.6	0.301^2^
Depression (%)	5.8	5.3	6.8	5.3	0.567^2^
Cancer (%)	3.2	2.9	3.7	2.6	0.846^2^
Parkinson’s disease (%)	0.0	0.0	0.0	0.0	n/a
COPD (%)	2.2	2.2	2.4	0.0	0.927^2^
Stroke (%)	0.3	0.0	0.8	0.0	0.068^2^
Myocardial infraction (%)	0.4	0.0	1.0	0.0	0.030^2^
Chronic kidney disease (%)	0.6	0.5	0.8	0.0	0.740^2^
HIV (%)	0.0	0.0	0.0	0.0	n/a
No. of diseases (mean ± SD)	0.35 ± 0.58	0.26 ± 0.53	0.47 ± 0.64	0.53 ± 0.69	0.001^4^
Multimorbidity (%)	4.6	2.9	7.3	5.3	0.004^2^

Follow-up					
Hypertension (%)	61.6	42.9	60.4	68.4	0.010^1^
Arthrosis (%)	26.1	20.4	25.1	29.8	0.197^1^
Type 2 diabetes mellitus (%)	25.8	26.5	26.1	24.7	0.903^1^
Depression (%)	17.9	18.4	18.3	16.7	0.838^1^
Cancer (%)	8.0	8.2	9.2	4.7	0.055^2^
Parkinson’s (%)	1.4	0.0	1.1	2.5	0.206^2^
COPD (%)	6.9	2.0	7.0	7.6	0.421^2^
Stroke (%)	3.6	8.2	2.8	4.7	0.065^2^
Myocardial infraction (%)	2.0	4.1	1.6	2.5	0.221^2^
Chronic kidney disease (%)	0.8	0.0	0.8	0.7	0.999^2^
HIV (%)	0	0	0	0	n/a
N° of diseases (media ± SD)	1.54 ± 1.26	1.31 ± 1.37	1.52 ± 1.23	1.63 ± 1.30	0.213^3^
Multimorbidity (%)	49.7	36.7	50.3	50.5	0.183^1^

N/A not applicable; COPD, chronic obstructive pulmonary disease.

* *p* value as determined with the chi square test^1^; Fisher’s exact test^2^; or ANOVA^3^.

Hypertension and low HDL-C was the most frequent morbidity combination, followed by diabetes and hypertension (Table 3). Women who had diabetes upon follow-up assessment, were over five times more likely to also have hypertension, and over two times more likely to have depression (Table 3).

**Table 3:** Multimorbidity and correlations between pairwise combinations of the selected diseases.

	Arthrosis		Hypertension		Diabetes		Depression	
	%	OR (95% CI)	%	OR (95% CI)	%	OR (95% CI)	%	OR (95% CI)
Arthrosis	–	–	20.9	3.31 (2.39−4.59)	8.3	1.49 (1.10−2.01)	7.8	2.68 (1.93−3.72)
Hypertension	20.9	3.31 (2.39−4.59)	–	–	22.0	5.14 (3.57−7.39)	14.0	2.56 (1.77−3.70)
Diabetes	8.3	1.49 (1.10−2.01)	22.0	5.14 (3.57−7.39)	–	–	6.4	1.78 (1.28−2.49)
Depression	7.8	2.68 (1.93−3.72)	14.0	2.56 (1.77−3.70)	6.4	1.78 (1.28−2.49)	–	–

Data are presented as percentages (%), odds ratios (OR) and confidence intervals (CI).

Diagnosed illnesses were retrieved from the SIGGES [8,9].

Based on baseline risk factors, we observed that obesity was the strongest risk factor for multimorbidity (OR 2.48, 95% CI, 1.71–3.61), followed by unqualified job (OR 2.18%, 95% CI, 1.67–2.83) and HDL-C <50 mg/dL (OR 1.31; 95% CI, 1.02–1.68). This regression model met the independence of errors assumption (Durbin–Watson: 1.96; ideal: 1.0–4.0). There was no collinearity, with a variance inflation factor ranging between 1.009 and 1.025. The Omnibus test reported a chi square equal to 64.950 (*p* < 0.0001). The Hosmer–Lemeshow test reported a chi square of 1.054 (*p* < 0.902).

## 4 Discussion

This cohort study showed that at baseline, when participants were 48 years old (mean), the prevalence of multimorbidity was 4.6%, figure that increased to almost 50% upon follow-up. This finding is in accordance with the fact that multimorbidity increases with age, or in other words, aging is accompanied with several chronic conditions [10].

Regarding multimorbidity prevalence, a review of 68 reports found that the frequency of multimorbidity in adult subjects was 33%, yet there were substantial prevalence differences between high- and middle/low-income countries (37% vs. 29%, respectively) [1].

The prevalence of multimorbidity found after follow-up in our study is smaller than the 71% reported in a systematic review of 11 studies conducted in high-income countries with populations within the same age range. This review pinpointed that the prevalence of multimorbidity is not only conditioned by age, but also by the number and type of diseases considered in the definition of multimorbidity, by geographic region, and whether the diseases information was self-reported or measured [11].

A cross-sectional study based on the World Health Organization SAGE survey conducted in low/middle-income countries showed a lower multimorbidity prevalence **(**60%**)** than that of high-income countries [12]. Our results are closer to those from low- and middle-income countries. Although Chile is classified as a high-income country, because of (large) within-country inequalities, this label should be interpreted cautiously; hence the similar profile to low and middle income countries. Another potential explanation for the multimorbidity prevalence herein reported is that our cohort included active working-class women, with regular access to healthcare. Under these circumstances, they could have received prevention or treatment, thus reducing the multimorbidity burden in this group [13].

Women in our cohort, who had multimorbidity at follow-up, had a different profile at baseline in comparison to their peers who were free of multimorbidity at follow-up: in the former group there was more obesity, hypertension, unqualified jobs, and lower HDL-C and higher triglyceride levels. This suggests that some of the more relevant risk factors for multimorbidity are modifiable on the long term (i.e. body mass index and lipid profile).

Regarding obesity, a large study with over 120 thousand men and women followed-up for 10 years in average, is also in agreement with our findings. In this study, the risk of multimorbidity in individuals with overweight was two-fold, and the risk among those with class I obesity was fifteen times higher [14]. Based on these international results, and on our estimates for Chilean women, increased body mass index (i.e. obesity) rises as a strong yet modifiable risk factor for multimorbidity.

Along with healthy lifestyles, effective treatment for some diseases are required to reduce the burden of multimorbidity. This would be the case of hypertension, which was strongly associated with multimorbidity upon follow-up in our sample. A study addressing 332 thousand subjects in England also reported hypertension as a very strong predictor for multimorbidity [15]. Hypertension prevention, along with securing effective treatment for these patients, would be needed to improve the overall health of the population, and reduce multimorbidity burden.

The role of estrogen deficit appears to be relevant. Menopause is associated with **a** higher risk of chronic conditions. For example, women with bilateral oophorectomy have shown a 24% higher risk of multimorbidity [16]. Similarly, compared to women who had menopause in their early 50 s, those with premature menopause had a 3-fold higher risk of multimorbidity in their 60 s [17]. Our baseline findings also signaled a higher rate of postmenopause among women with multimorbidity.

Education also appears to be a relevant risk factor for multimorbidity. For instance, our study found that individuals with unqualified jobs (in general with lower education) had a 40% higher probability of developing multimorbidity at follow-up. This finding is in agreement with one study reporting that individuals having a bachelor’s degree have a lower risk of multimorbidity than those with lower education (32% vs. 60%) [18].

In our cohort, the prevalence of diabetes increased from 2.3% to 26% between baseline and follow-up. However, we did not find any differences regarding baseline diabetes status among women who developed multimorbidity and those who did not. This is interesting because diabetes is a pro-inflammatory condition associated with several other chronic diseases [19]. This preliminary finding may suggest that not all diabetic patients are at higher risk of multimorbidity, indicating that there may be some diabetes clusters with higher/lower risk. Likewise, another cohort study signaled hypertension, obesity and smoking as multimorbidity risk factors; nonetheless, diabetes was not identified as a risk factor [15].

Dyslipidaemia at baseline, such as low HDL-C or high triglyceride levels, were associated with higher multimorbidity during follow-up. Cross-sectional evidence has supported the relevance of dyslipidaemia in multimorbidity. In this sense, the National Health and Nutrition Examination Survey 2007–2012 showed that women with low HDL-C had twice the prevalence of multimorbidity than women with normal HDL-C [20].

In our study, the most frequent components of multimorbidity were hypertension, arthrosis, diabetes and depression. Comparing our estimates with other studies is difficult because of different epidemiological profiles between populations and used analytical methodologies. Nonetheless, a systematic review of studies in high-income countries of individuals aged 65 and above, showed a similar pattern regarding frequency and ranking; however, they also included dyslipidaemia at the second place of the ranking [11]. Regardless of socioeconomic status at the country level, multimorbidity, and its components, appear to be a global health issue.

In our study population, the most prevalent pairwise combinations of diseases were diabetes and hypertension, followed by arthrosis and depression. Researchers in Germany have reported a similar profile [21].

Regarding the strengths of our study one can mention its longitudinal cohort design that specifically evaluated the long-term impact over aging of risk factors observed in women during middle-age. On the other hand, our study used standardized definitions for specific morbidities in a very homogeneous female population. Among limitations, one can mention the fact that the cohort only included civil servants. This group could have had better access to healthcare than the general population, fact that constitutes a selection bias.

In conclusion, in this cohort of Chilean women, multimorbidity was highly prevalent in the elderly; also, the most common multimorbidity pairwise combination was hypertension and diabetes. Obesity during mid-life was the strongest predictor of multimorbidity at elder age, followed by unqualified job, and low HDL-C levels. These estimates are relevant for Chile and other countries with similar health and population profiles. Indeed, these results can inform about high-risk groups who should receive attention to reduce the burden of multimorbidity.

## Contributors

Juan E. Blümel was responsible for conceptualization, methodology, and drafting off the article.

Rodrigo M. Carrillo-Larco contributed to revision of the article, translation and editing.

María S. Vallejo was responsible for statistical analysis and revision of the article.

Peter Chedraui contributed to revision of the article, translation and editing.

## Conflict of interest

Juan E. Blümel has received financial support from Grünenthal for attendance at the International Menopause Society (IMS) Congress Vancouver, Canada, 2018 and at the European Menopause and Androgen Society (EMAS) Congress, Berlin, Germany, 2019.

Rodrigo Carrillo-Larco, María S. Vallejo and Peter Chedraui declare they have no conflicts of interest.

## Funding

This research did not receive any specific grant from funding agencies in the public, commercial, or non-for-profit sectors. Rodrigo M. Carrillo-Larco is supported by a Wellcome Trust International Training Fellowship grant (214185/Z/18/Z). Peter Chedraui is supported by the Sistema de Investigación y Desarrollo (SINDE) and the Vice-Rectorado de Investigación & Postgrado (VRIP) of the Universidad Católica de Santiago de Guayaquil, Guayaquil, Ecuador, through grant No. SIU-318-853-2014 (The Omega II, Women’s Health Project).

## Ethics

The study was approved by the local ethics committee (Southern Metropolitan Health Service, Santiago de Chile, Chile) and was in complete agreement with the Declaration of Helsinki. All patients provided written informed consent.

## Data sharing and collaboration

Data for multimorbidity in a cohort middle-aged women: Mendeley Data:

## Provenance and peer review

This article has undergone peer review.
